# Risk factors for wound complications after associating liver partition and portal vein ligation for staged hepatectomy (ALPPS) compared to repeated liver resection - a propensity score matching analysis

**DOI:** 10.1007/s00423-024-03540-4

**Published:** 2024-11-13

**Authors:** Elias Khajeh, Nastaran Sabetkish, Ali Ramouz, Alexander Werba, Rosa Klotz, Christoph W. Michalski, Arianeb Mehrabi, Frank Pianka

**Affiliations:** 1 Department of General, Visceral and Transplantation Surgery, University Hospital Heidelberg, Im Neuenheimer Feld 420, 69120 Heidelberg, Germany; 2 Study Center of the German Surgical Society (SDGC), Heidelberg, Germany

**Keywords:** Incisional hernia, Surgical site infection, Wound healing, Liver surgery

## Abstract

**Aim:**

Sufficient liver function is crucial in extracellular matrix growth, hemostasis, and wound healing. Repeated abdominal surgery is a known risk factor for the development of wound complications. This study aimed to evaluate this high-risk constellation in patients undergoing associated liver partition and portal vein ligation for staged hepatectomy (ALPPS) and repeated liver resections (RLR) in comparison to single liver resection (SLR).

**Method:**

Forty patients who underwent ALPPS between 2011 and 2020 were evenly matched with patients undergoing RLR or SLR (*n* = 40 per group) using propensity scores. Postoperative outcomes were compared and factors associated with wound complications were analyzed.

**Results:**

Postoperative wound complications were significantly more frequent in ALPPS group (*p* = 0.001). The reoperation rate was not significantly different between the three groups (*p* = 0.143). However, surgical reintervention due to wound complications occurred more frequently in the ALPPS group in relation to RLR and SLR (17.5% vs. 7.5% and 5% respectively). Length of stay was significantly longer in the ALPPS group (*p* = 0.033). ALPPS was an independent risk factor for postoperative wound complication (OR = 8.55, 95% CI:1.07–68.44, *p* = 0.043). Risk factor analysis identified age ≥ 60 years (OR = 27.64, 95% CI:3.09-246.75, *p* = 0.003), BMI ≥ 30 kg/m^2^ (OR = 30.21, 95% CI:3.35-271.83, *p* = 0.002), and low postoperative albumin levels (OR = 168.41, 95% CI:7.76-3651.18, *p* = 0.001) as independent predictors of postoperative wound complications after major liver resection.

**Conclusion:**

Patients undergoing ALPPS and RLR are faced with a high risk of developing wound complications. Older age, obesity, a history of previous abdominal surgery, and a decreased postoperative albumin level were independent risk factors for wound complications.

## Introduction

While surgery remains the keystone in the treatment of liver malignancies [1], the risk of recurrence for primary or secondary liver malignancies is still as high as up to 40% with a growing patient population in need of extensive or repeated liver resections [2]. In the past two decades, recent surgical advancements have made staged hepatectomy or repeated liver resections an option in patients with otherwise unresectable liver tumors or recurrent disease [3]. Patients with potentially insufficient future liver remnant (FLR) are candidates for associating liver partition and portal vein ligation for staged hepatectomy (ALPPS) which has been developed since 2007 to promote an accelerated FLR growth within 6–9 days [4]. Although this technique has proven advantageous in achieving an R0 resection with a reduced risk of post-hepatectomy liver failure (PHLF), it still has a high risk of morbidity and mortality [5, 6]. Wound complications such as fascial dehiscence, incisional hernia, or surgical site infection (SSI) are common in patients undergoing major liver surgery. However, only little is known about wound complication rates and associated factors after the ALPPS procedure.

Impaired liver function and repeated hepatic resections are important risk factors causing postoperative complications after major liver resection [7–9]. Major surgeries are generally associated with a diminishing subcutaneous collagen accumulation resulting in a reduced systemic wound-healing capacity [10]. Repeated abdominal incisions and a history of previous surgery are also known risk factors for postoperative wound complications [11]. Repeated incisions in combination with impaired liver function may affect wound healing at the highest level [12–14]. As a result, patients undergoing either ALPPS or repeated metastasectomy may be confronted with postoperative surgical site infection (SSI) or fascial dehiscence, due to impaired wound healing. However, it is not known if performing repeated incisions in a short period, as in the ALPPS procedure, is an additional risk factor compared to repeated liver resections in longer intervals.

The present study aimed to evaluate the occurrence of postoperative wound complications in patients undergoing ALPPS in comparison to repeated liver resections (RLR) for cancer recurrence after major liver resection and patients after a single major liver resection (SLR). Furthermore, we aimed to assess risk factors for surgical wound complications after repeated liver surgeries.

## Materials and methods

### Study population

**Fig. 1:**
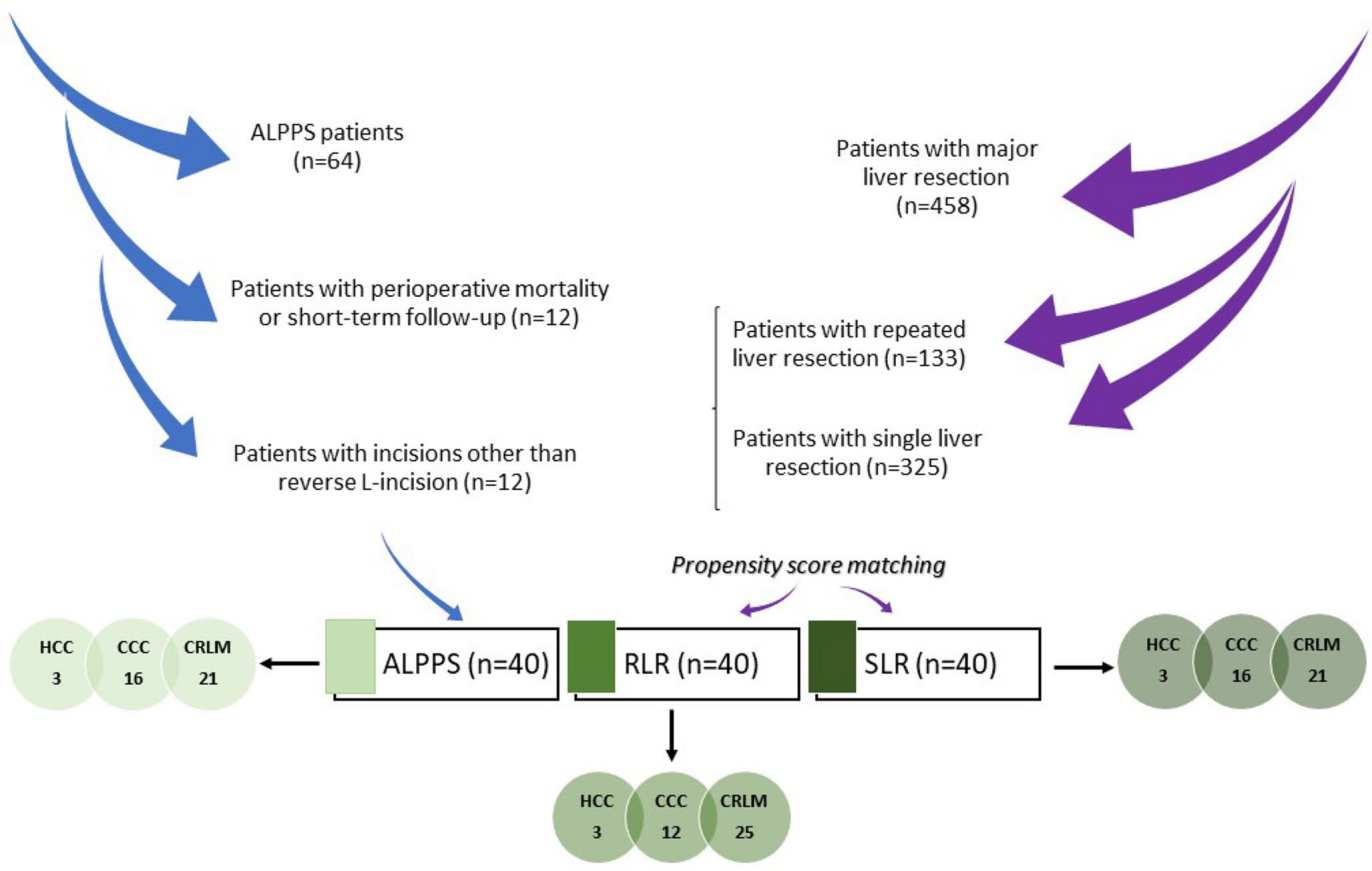
Patients allocation in different major liver resection techniques after propensity score matching

The data of 64 consecutive patients who underwent major liver resection through an ALPPS procedure between October 2011 and September 2020 at the Department of General, Visceral, and Transplantation Surgery of the University Heidelberg were extracted from our prospectively maintained database. Inclusion criteria were age > 18 years, liver resection using a reverse L-shaped incision (rL-incision), and a minimum follow-up of 12 months. After exclusion of patients with perioperative mortality (up to 30 days) or short-term follow-up (*n* = 12), and those with incisions other than rL-incision (*n* = 12), data of 40 patients were analyzed. Data from adult patients with major open liver resection using an rL-incision during the same period were also collected (*n* = 458). Within this group, 133 patients underwent a repeat liver resection (RLR) due to recurrence, while the remaining 325 patients underwent a single liver resection (SLR) without any subsequent liver surgery. Propensity-score matching was meticulously performed to achieve a 1:1:1 ratio between patients in the ALPPS, RLR, and SLR groups, using gender, age, and the indication for surgery serving as matching variables (see Fig. 1).

The study was approved by the regional ethics committee of the medical faculty (approval number: S-754/2018). The study was conducted following the STROCSS 2021 reporting guidelines and the declaration of Helsinki.

### Patient data collection and measurements

Baseline demographic and clinical data including age, gender, body mass index (BMI), history of diabetes mellitus (DM), previous abdominal operations, diagnosis, preoperative laboratory data on the day before the surgery (albumin, bilirubin, and the international normalized ratio [INR]) as well as *intra- and postoperative data* (including the extent of resection, operation time, length of hospital stay, postoperative complications, re-operation, and length of follow-up) were extracted from a prospective liver surgery database.

Wound complication was defined as the occurrence of one of the following: SSI, wound dehiscence, abdominal fascial dehiscence, or incisional hernia. SSI was defined according to Centers for Disease Control and Prevention (CDC) criteria [15, 16]. Length of follow-up started from the second performed surgery in ALPPS and RLR groups. To assess the role of obesity in postoperative outcomes, a subgroup analysis was performed for patients with a BMI ≥ 30 kg/m².

### Statistical analysis

Statistical data analysis was performed with SPSS software (Version 25, SPSS Inc., Chicago, IL, USA). Categorical data are presented as proportions and percentages, and continuous data as the median and interquartile range (IQR). Categorical data are compared using the chi-square test of association or Fisher’s exact test. Continuous data are compared using one-way analysis of variance (ANOVA) followed by the Tukey posthoc test or Kruskal-Wallis H test. Propensity score-matching analysis was used to build a matched group of patients and minimize differences in confounders and baseline characteristics between the patients using age, gender, and surgical indication. The propensity score matching algorithm was performed using R, version 3.5.1 (The R Foundation for Statistical Computing, Vienna, Austria). Logistic regression analysis was used to estimate the propensity score. Patients were matched based on the logit of the propensity score by selecting the nearest available matching using a caliper width of 0.1 of the standard deviation of the estimated propensity score between study groups. Accordingly, a 1:1 matched analysis was conducted. Logistic binary regression analysis was used to calculate the multivariate odds ratios (OR) and 95% confidence intervals (95% CI). Variables with *p* < 0.2 in the univariate analysis were entered into the multivariate regression analysis. All statistical tests were two-sided and a *p* < 0.05 was considered significant.

## Results

### Perioperative data

In total, 40 patients with a median age of 59.5 years (IQR = 11.75) undergoing ALPPS for various malignant diagnoses met the inclusion criteria within the study period. Another 40 matched patients with a median age of 59.0 years (IQR = 12.0) were included in the RLR group. The second control group included 40 individuals with a median age of 60.2 years (IQR = 14.0) who underwent an SLR. The majority of patients were male (*n* = 81, 67.5%) and the median BMI was 24.7 kg/m^2^. The baseline, intraoperative, and postoperative characteristics of patients in each group are summarized in Table 1. Between the subgroups, there were no significant differences in preoperative characteristics including age, gender, BMI, DM, previous abdominal surgery, the indication of surgery, preoperative albumin, bilirubin, and INR levels.

**Table 1:** Baseline characteristics and surgical data of patients undergoing major hepatectomy with single or multiple resections

	ALPPS (*n* = 40)	RLR (*n* = 40)	SLR (*n* = 40)	*P*-value
Age (year) median (Q1, Q3)	59.5 (53.2, 65.7)	59.0 (52.0, 64.0)	60 (52, 66)	0.549
Gender (female) n (%)	12 (30.0)	14 (35.0)	13 (32.0)	0.892
BMI median (Q1, Q3)	25.5 (23.0, 29.5)	24.2 (22.0, 30.3)	25 (22.1, 28,8)	0.732
Diabetes n (%)	14 (35)	7 (17.5)	6 (15.0)	0.066
Previous abdominal surgery n (%)	18 (45.0)	15 (37.5)	11 (27.5)	0.265
Indication n (%)				
HCC	3 (7.5)	3 (7.5)	3 (7.5)	0.877
CCC	16 (40.0)	12 (30.0)	16 (40.0)	
CRLM	21 (52.5)	25 (62.5)	21 (52.5)	
Preoperative Albumin median (Q1, Q3)	40.3 (37.7, 45.2)	42.4 (39.5, 44.0)	42.5 (38.9, 44.2)	0.750
Preoperative Bilirubin median (Q1, Q3)	0.7 (0.5, 1.6)	0.6 (0.4, 1.5)	1.4 (0.8, 1.6)	0.562
Preoperative INR median (Q1, Q3)	1.0 (0.9, 1.0)	1.0 (0.9, 1.0)	1.0 (0.9, 1.1)	0.281
Resection type n (%)				
Extended hepatectomy	31 (77.5)	13 (32.5)	10 (25.0)	**< 0.001** ^⁑^
Hemihepatectomy	9 (22.5)	27 (67.5)	30 (75.0)	
Operation time for stage 1 (min) median (Q1, Q3)	240.0 (204.7, 326.2)	201.5 (165.0, 240.0)	242.5 (182.5, 273.7)	**0.020** ^*^
Operation time for stage 2 (min) median (Q1, Q3)	128.0 (95.0, 195.0)	202.5 (150.0, 260.7)	-	**0.001**
Postoperative Albumin median (Q1, Q3)	27.5 (23.4, 30.8)	31.1 (28.2, 33.4)	30.8 (26.3, 35.1)	**0.005** ^*§^
Postoperative Bilirubin median (Q1, Q3)	1.5 (0.9, 2.3)	1.3 (0 8, 2.3)	0.9 (0.7, 1.5)	**0.029** ^§^
Postoperative INR median (Q1, Q3)	0.9 (0.5, 1.9)	1.2 (1.1, 1.3)	1.15 (1.11, 1.2)	0.134
Overall wound complications n (%)	15 (37.5)	9 (22.5)	5 (12.5)	**0.032** ^*§^
In-hospital wound complications n (%)	6 (15.0)	2 (5.0)	1 (2.5)	0.485
Reoperation for wound complication n (%)	7 (17.5)	3 (7.5)	2 (5.0)	0.143
Total postoperative complications n (%)	15 (37.5)	6 (15)	4 (10)	**0.005** ^*§^
Total ICU stays (day) median (Q1, Q3)	0	0	0	0.064
Total IMC stays (day) median (Q1, Q3)	6.5 (0, 10.7)	0 (0, 2)	0 (0, 2)	**< 0.001** ^*§^
Total hospital stays (ALPPS – first stage) (day) median (Q1, Q3)	29.0 (24.2, 36.0)	14.0 (9.0, 21.0)	13.0 (9.0, 23.2)	**< 0.001** ^*§^
Total hospital stays (ALPPS – second stage) (day) median (Q1, Q3)	20 (16.0, 20.0)	14.0 (9.0, 21.0)	13.0 (9.0, 23.2)	**0.001** ^§^
Follow-up (month) median (Q1, Q3)	26.0 (17.2, 41.7)	22.5 (15.2, 35.5)	23.5 (16.0, 33.0)	0.258

Q1: Interquartile 1; Q3: Interquartile 3; ALPPS: Associating liver partition and portal vein ligation for staged hepatectomy; RLR: repeated liver resection; SLR: single liver resection; BMI: Body mass index; HCC: Hepatocellular carcinoma; CCC: Cholangiocarcinoma; CRLM: Colorectal liver metastasis; INR: International normalized ratio; SSI: surgical site infection

^*^ Significant difference between ALPPS and RLR, ^§^ Significant difference between ALPPS and SLR ^⁑^ Significant difference between RLR and SLR

The operation duration was significantly different among these three groups (*p* = 0.001). Pairwise comparisons of the mean operation times revealed a significantly longer operation of the first stage of ALPPS compared to the RLR group (mean difference = 71.27 min, SE = 19.19, *p* < 0.001). Comparison between ALPPS and SLR (0.125) as well as RLR and SLR (*p* = 0.193) revealed no significant difference. However, when comparing the operation duration of the second stage of ALPPS, the surgical procedure was longer in the RLR group compared to ALPPS (213 vs. 150 min, *p* = 0.001).

### Postoperative data

#### Wound outcomes

**Fig. 2:**
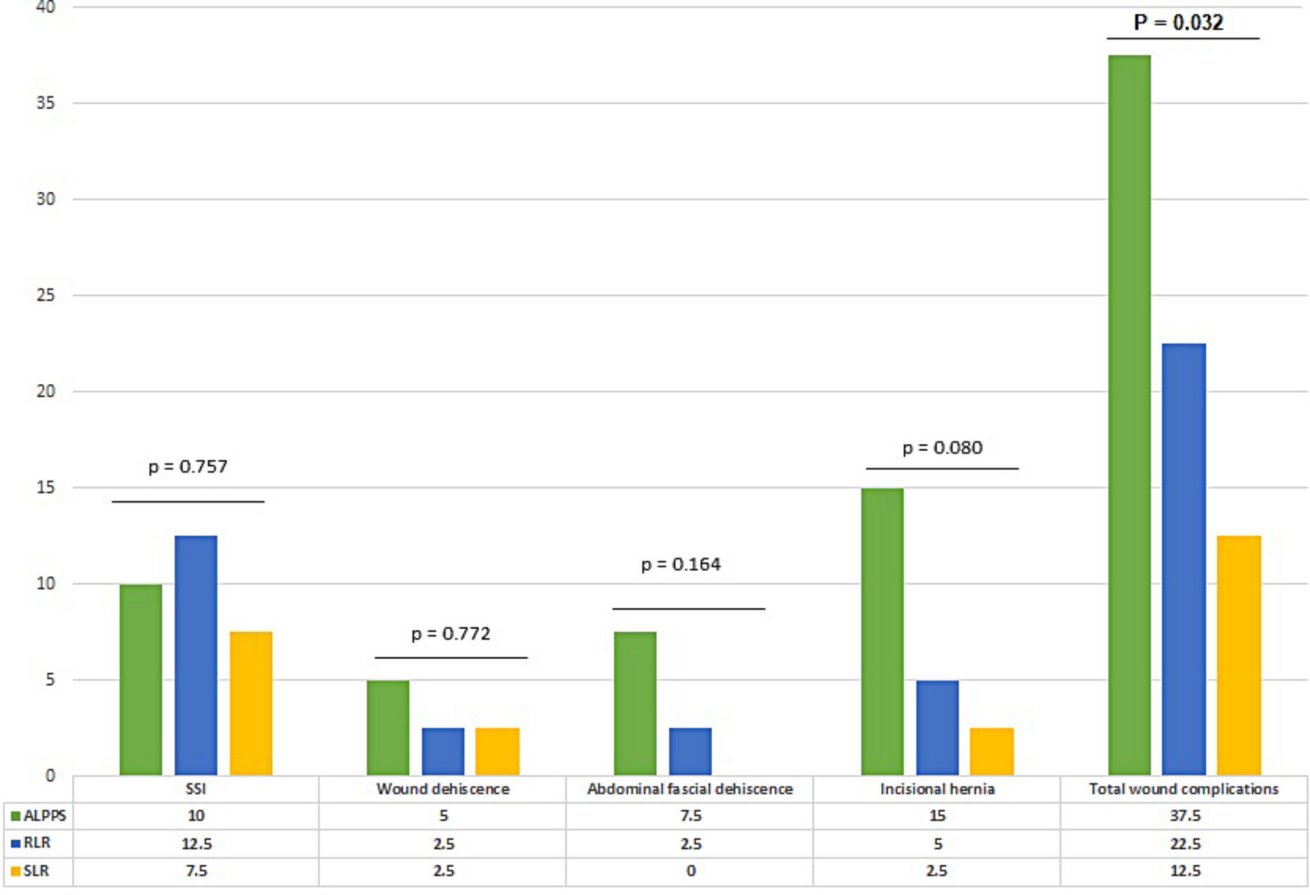
Wound outcomes in different major liver resection techniques

Overall wound complications were significantly higher in the ALPPS group (37.5%) compared to RLR and SLR groups (22.5% and 12.5%, *p* = 0.032). However, when studying different subgroups of wound complication, the statistical analysis was not different among three surgical groups (*p* = 0.757 for SSI, *p* = 0.772 for wound dehiscence, *p* = 0.164 for abdominal fascial dehiscence, and *p* = 0.080 for incisional hernia). The most frequent complication was incisional hernia in ALPPS groups (15.0%); while SSI was the most common wound complication in RLR and SLR groups (12.5% and 7.5% respectively) (Fig. 2). There was no case of organ-space SSI in any group and all patients with superficial SSI were managed by antibiotic therapy. Our statistical analysis revealed no significant difference in terms of in-hospital or late complication occurrence.

The overall mean interval between surgery and wound complication occurrence was 9.4 ± 16.45 months. When considering the short-term complications (in-hospital), 4 (10.0%), 5 (12.5%), and 3 (7.5%) patients had SSI in ALPPS, RLR, and SLR groups, respectively. Additionally, 2 (5.0%), 1 (2.5%), and 1 (2.5%) patients suffered wound dehiscence in the above-mentioned groups. Furthermore, fascial dehiscence occurred in 3 (7.5%) patients in the ALPSS and 1 (2.5%) patient in the RLR group during their hospital stay. We considered late complications as those occurring after the hospitalization that occurred in 6 (15.0%), 2 (5.0%), and 1 (2.5%) patients in the ALPPS, RLR, and SLR groups, respectively. The need for reoperation due to wound complications was highest in the ALPPS group (17.5% vs. 7.5% and 5.0% in the RLR and SLR, respectively).

#### Laboratory findings

The postoperative albumin levels were significantly different among the three groups (*p* = 0.005). A pairwise comparison revealed a significantly lower albumin level in ALPPS patients as compared to the RLR group (mean difference=-3.05, SE = 1.15, *p* = 0.025). Similarly, the albumin level was lower in ALPPS as compared to SLR (mean difference=-3.13, SE = 1.15, *p* = 0.021). There was no significant difference in terms of postoperative albumin level between the RLR and SLR groups (*p* = 0.997). The postoperative bilirubin level was significantly different among these three groups (*p* = 0.029). Pairwise comparisons showed a significant difference between the ALPPS and SLR groups (mean difference=-0.47, SE = 0.18, *p* = 0.034). Comparison between ALPPS and RLR (0.845) and RLR and SLR (*p* = 0.1243) revealed no significant difference in postoperative bilirubin level. Postoperative INR levels were not statistically significant among groups (*p* = 0.455). The median interval between the two surgeries in the RLR group was 9 months (range 6 to 59 months).

#### Overall complications

Statistical analysis revealed a significantly higher rate of postoperative total complications in ALPPS compared to the other two groups (*p* = 0.005). 7.5%, 5%, and 25% of patients in the ALPPS group had a postoperative biliary leakage, bleeding, or non-surgical complication. The rate of these complications in the RLR group was 5%, 2.5%, and 7.5%, respectively. However, 5%, 2.5%, and 4.5% of patients in the SLR group had the above-mentioned postoperative complications.

#### Intensive care and hospital-stay

Even though ICU stay was similar among groups, IMC stay was significantly higher in patients who underwent ALPPS (*p* < 0.001). Pairwise comparisons showed a higher IMC stay in ALPPS compared to both the SLR (mean difference = 4.82, SE = 0.81, *p* < 0.001) and RLR (mean difference = 5.35, SE = 0.81, *p* < 0.001) groups. Furthermore, statistical analysis revealed no significant difference in the length of IMC stay between the RLR and SLR groups in terms of hospital stay (*p* = 0.798). The median duration of the follow-up was 22.5 months (range: 12–102 months). There was no significant difference in terms of follow-up duration among the groups (*p* = 0.190). The hospital stay was significantly different among these three groups when considering the second stage of ALPPS as the initial day of hospitalization in this subgroup (20, 14, and 13 days in the ALPPS, RLR, and SLR groups, respectively, *p* < 0.001). Our analysis revealed a significantly longer hospital stay in the ALPPS as compared to the SLR group (mean difference = 9.47, SE = 3.82, *p* = 0.039). However, the result of our statistical analysis revealed no significant difference neither between the ALPPS and RLR groups (*p* = 0.093) nor between the RLR and SLR groups (*p* = 0.932) concerning hospital stay. Similarly, patients were hospitalized in the ALPPS compared to the SLR group (mean difference = 17.65, SE = 3.80, *p* < 0.001). However, no significant difference was detected between the RLR and SLR groups in terms of hospital stay (*p* = 0.951).

### Factors associated with postoperative wound complications

Univariate analysis of predictive factors of postoperative wound complications revealed that age ≥ 60 (< 0.001), BMI ≥ 30 (*p* < 0.001), DM (*p* < 0.001), previous abdominal surgery (*p* < 0.001), ALPPS (*p* = 0.013), preoperative bilirubin level (0.178), and postoperative albumin levels (*p* < 0.001) were significantly correlated with postoperative wound complications. Multivariable regression analysis was performed on these six variables. Age ≥ 60 years (OR = 27.64, 95% CI: 3.09-246.75, *p* = 0.003), BMI higher than 30 kg/m2 (OR = 30.21, 95% CI: 3.35-271.83, *p* = 0.002), undergoing ALPPS surgery (OR = 8.55, 95% CI: 1.07–68.44, *p* = 0.043), and decreasing of postoperative albumin to less than 30 g/dL (OR = 168.41, 95% CI: 7.76-3651.18, *p* = 0.001) were identified as independent predictors of posthepatectomy wound complications (Table 2).

**Table 2:** Univariate and multivariate analysis of predictive factors of postoperative wound healing complications after major hepatectomy with single or multiple resections

	Univariate			Multivariate		
	OR	95% CI	*p*	OR	95% CI	*p*
Age, years (≥ 60)	**7.68**	**2.68–21.99**	**< 0.001**	**27.64**	**3.09-246.75**	**0.003**
BMI, kg/m^2^ (≥ 30)	**7.79**	**2.97–20.41**	**< 0.001**	**30.21**	**3.35-271.83**	**0.002**
Gender	1.09	0.44–2.68	0.847	-	-	-
Diabetes	**8.95**	**3.40-23.51**	**< 0.001**	4.52	0.76–26.79	0.096
Previous abdominal surgery	**7.76**	**3.02–19.90**	**< 0.001**	3.71	0.57–24.15	0.170
Surgery type (compared to SLR)						
ALPPS	**4.20**	**1.35–13.06**	**0.013**	**8.55**	**1.07–68.44**	**0.043**
RLR	2.03	0.61–6.71	0.245	2.81	0.31–24.81	0.351
Resection type (Extended)	0.90	0.40–2.19	0.948	-	-	-
Operation time, min (≥ 180)	0.61	0.26–1.41	0.251	-	-	-
Preoperative Albumin, g/L (< 40)	1.12	0.47–2.67	0.792	-	-	-
Preoperative Bilirubin, mg/dL (> 1)	**0.55**	**0.23–1.31**	**0.178**	0.71	0.12–4.01	0.698
Preoperative INR (> 1.1)	0.93	0.23–3.65	0.923	-	-	-
Postoperative Albumin, g/L (< 30)	**63.00**	**8.16-486.31**	**< 0.001**	**168.41**	**7.76-3651.18**	**0.001**
Postoperative Bilirubin, mg/dL (> 1)	1.34	0.57–3.15	0.501	**-**	**-**	-
Postoperative INR (> 1.1)	1.35	0.52–3.41	0.517	-	-	-

OR: Odds ratio; BMI: Body mass index; ALPPS: Associating liver partition and portal vein ligation for staged hepatectomy; RLR: repeated liver resection; INR: International normalized ratio

* For ALPPS and RLR the operation time for the second stage was considered

### Subgroup analysis in patients with obesity

**Fig. 3:**
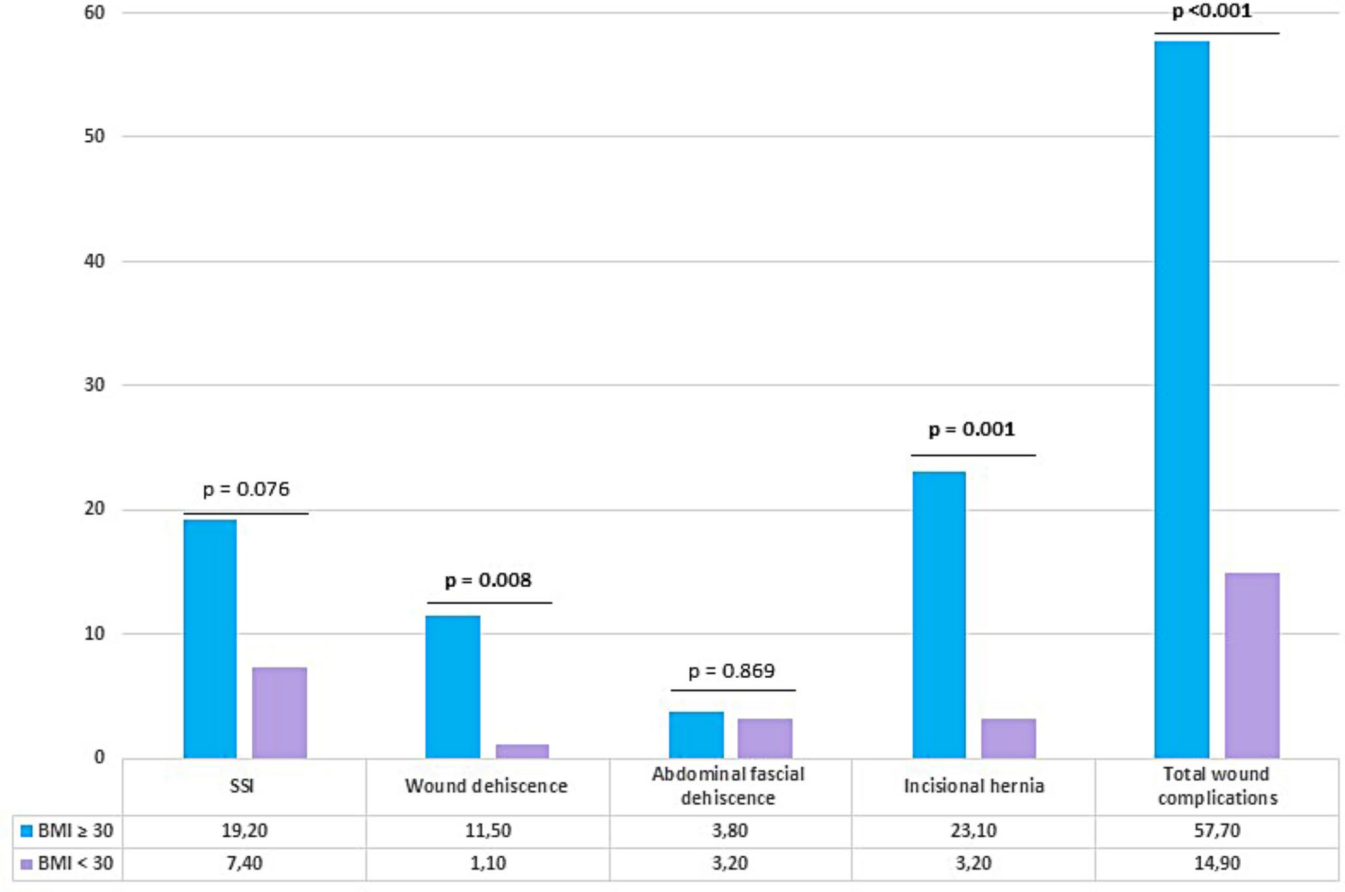
Wound outcomes in patients with and without obesity

A total of 26 patients had a BMI ≥ 30 kg/m² of whom 8 (30.8%), 10 (38.5%), and 8 (30.8%) patients underwent ALPPS, RLR, and SLR, respectively. Results of the subgroup analysis revealed no significant difference in preoperative characteristics in patients with and without obesity except diabetes which was significantly higher in patients with obesity (*p* = 0.028). Intraoperative variables were similar in both groups. The evaluation of postoperative wound complications is depicted in Fig. 3. Similarly, wound dehiscence (*p* = 0.008), incisional hernia (*p* = 0.001), and total wound complications (*p* < 0.001) were significantly higher in patients with a BMI ≥ 30 kg/m². Additional need for reoperation caused by wound complications was significantly higher in patients with obesity (*p* = 0.001).

## Discussion

In this study, patients undergoing ALPPS, repeated, and non-repeated major liver resections were compared for the first time regarding short- and long-term wound complications with a special focus on determining risk factors. Analyses showed that wound complications were significantly higher in patients undergoing ALPPS followed by RLR and SLR. High age (older than 60 years old), obesity (BMI higher than 30 kg/m^2^), undergoing ALPPS, and lower postoperative albumin were identified as independent risk factors for post-hepatectomy wound complications.

The underlying cause of wound complications is known to be multifactorial [17]. However, age, obesity, DM, surgical technique, method of closure, surgical complications, immunosuppressive therapy, infectious postoperative complications, and repeated surgical interventions have been determined as potent postoperative risk factors, influencing the development of post-surgical abdominal wound complications [18, 19]. Relaparotomies are believed to carry a higher risk for long-term complications such as incisional hernia [20]. Adhesiolysis, which is commonly performed in re-laparotomies, is believed to increase the risk of intraabdominal injuries, wound infections, and length of hospital stay. Furthermore, this procedure leads to a twofold risk of incisional hernia, increasing costs and reducing the quality of life [13, 20, 21]. Similarly, the present study revealed a significantly higher prevalence of wound complications in patients undergoing ALPPS and RLR. The multivariable regression analysis showed that ALPPS is an independent predictor of wound healing complications in patients undergoing major liver resection.

Results of a recent study revealed that patients’ age and preoperative albumin, as well as operative time, could be considered significant predictors of surgical morbidity [22]. Similar results were obtained in a recent study by Wong et al., indicating that the albumin-bilirubin ratio was a precise predictor of severe posthepatectomy liver failure and 30-day mortality in patients undergoing hepatectomy [23]. The presented multivariable regression analysis results show that low postoperative albumin is an independent predictor of wound healing complications in patients undergoing major liver resection.

Previous laparotomy and disturbed wound healing were found to be of significant influence on the development of incisional hernia [24]. Although the majority of patients with postoperative abdominal wall complications could be managed with less invasive modalities such as antibiotics and simple dressings, a considerable number of cases may need additional intervention with up to 25% of patients needing reoperation in a collective undergoing renal transplantation [25]. However, the results of the multivariate analysis revealed that previous abdominal surgery may not be considered as a risk factor for wound complications in these patients.

For patients who are at a high risk of sustaining a significant abdominal wall complication, both perioperative and postoperative managements could be amended in a way to prevent the occurrence of wound complications and/or further abdominal wall dehiscence. The effectiveness of prophylactic mesh reinforcement in the reduction of incisional hernia rate has been previously discussed [26]. Results of a recent study after kidney transplantation revealed that mesh reinforcement decreases SSI and overall wound complications [27]. Promising outcomes in reducing parastomal hernia with mesh placement have also been reported [28]. The use of biomaterials for abdominal wall repair has markedly modified the postoperative course of wound healing in the last few decades resulting in a reduction in recurrence rates, from up to 50% to less than 20% [29, 30]. Another preventive modality may be negative pressure wound therapy (NPWT) which is applied for complex soft tissue wounds and affects the reconstruction process by shortening the healing time [31].

To date, a notable gap in the existing literature is the absence of studies examining wound complications in patients undergoing ALPPS. Furthermore, we conducted a comparative analysis of postoperative wound complications between ALPPS, RLR, and SLR.

Our findings revealed distinct characteristics associated with ALPPS. Specifically, ALPPS was associated with a lengthier duration of the first-stage operation, elevated postoperative levels of albumin and bilirubin, an increased incidence of overall wound complications, and consequently, prolonged total hospital stays. Furthermore, our analysis indicated that older patients with higher Body Mass Index (BMI) undergoing ALPPS, particularly those with reduced postoperative albumin levels, may be at a heightened risk of experiencing postoperative wound healing complications.

Nevertheless, this study has several limitations, primarily owing to its retrospective nature and inherent heterogeneity within the study groups. To mitigate this, we employed propensity-matching techniques, carefully selecting comparable groups based on age, gender, and surgical indication. It is worth noting that the variability in skin, subcutaneous tissue, and fascial closure techniques employed during the study period may introduce some statistical uncertainty. Although we limited our analysis to patients undergoing major hepatectomies, heterogeneity remains in the extent of liver resection across groups, particularly for ALPPS. Additionally, factors such as pre-existing comorbidities and nutritional status, which are known to influence wound healing and postoperative outcomes, may still contribute to residual confounding despite propensity score matching.

Addressing the need for more comprehensive investigations, further studies are warranted to bridge the knowledge gap concerning the standard of care and the consequences of relaparotomies. Nonetheless, it is important to highlight that the presented ALPPS cohort stands as the most extensive to date and offers crucial insights into wound complications, potential risk factors, and strategies for prevention.

In conclusion, while ALPPS remains a compelling surgical option for major liver resections in patients with previously unresectable tumors, it does pose an elevated risk of wound complications when compared to SLR and RLR procedures. Independent risk factors identified for post-hepatectomy wound complications include older age, obesity, the utilization of ALPPS as the surgical technique, and lower postoperative albumin levels.

Consequently, meticulous patient selection is advised to enhance postoperative outcomes, particularly in high-risk procedures such as ALPPS and among patients undergoing RLR.

## Data Availability

No datasets were generated or analysed during the current study.
